# Low-Field Nuclear Magnetic Resonance Characteristics of Biofilm Development Process

**DOI:** 10.3390/microorganisms9122466

**Published:** 2021-11-29

**Authors:** Yajun Zhang, Yusheng Lin, Xin Lv, Aoshu Xu, Caihui Feng, Jun Lin

**Affiliations:** 1 College of Instrumentation and Electrical Engineering, Jilin University, Changchun 130061, China; yajun15@mails.jlu.edu.cn (Y.Z.); linys19@mails.jlu.edu.cn (Y.L.); xinlv15@mails.jlu.edu.cn (X.L.); xuks15@mails.jlu.edu.cn (A.X.); 2 Key Laboratory of Geophysics Exploration Equipment, Ministry of Education of China, Changchun 130061, China

**Keywords:** low-field NMR, biofilm growth, bacterial cell, T_2_ relaxation, porous media

## Abstract

To in situ and noninvasively monitor the biofilm development process by low-field nuclear magnetic resonance (NMR), experiments should be made to determine the mechanisms responsible for the T_2_ signals of biofilm growth. In this paper, biofilms were cultivated in both fluid media and saturated porous media. T_2_ relaxation for each sample was measured to investigate the contribution of the related processes to T_2_ relaxation signals. In addition, OD values of bacterial cell suspensions were measured to provide the relative number of bacterial cells. We also obtained SEM photos of the biofilms after vacuum freeze-drying the pure sand and the sand with biofilm formation to confirm the space within the biofilm matrix and identify the existence of biofilm formation. The T_2_ relaxation distribution is strongly dependent on the density of the bacterial cells suspended in the fluid and the stage of biofilm development. The peak time and the peak percentage can be used as indicators of the biofilm growth states.

## 1. Introduction

Hydrocarbons, heavy metals, and microplastics are serious environmental problems with which governments around the world are concerned. The native microbes can be artificially activated by injecting nutrients to form a biobarrier to prevent pollutants’ migration or to promote pollutants’ degrading rate in contaminated soil and aquifers as environmentally friendly, low-cost, and effective methods [1,2,3,4,5,6]. During these processes, the formation of biofilms is crucial for accelerating pollutants’ adsorption and degradation [2,3,7]. The microbe cells secrete a massive extracellular polymeric substance (EPS) and are arranged all over the viscous EPS matrix in an optimal way to capture resources and pollutants and construct a dynamic biofilm system [8].

Efforts have been made by researchers to investigate the factors that impact biofilm development and fate with experimental tests and mathematical simulations [9,10,11,12,13]. Models that simulate biofilm systems should depend on fundamental laws (first principles) such as reaction/transport principles, fluid mechanics, and structural mechanics [14]. The models also need to take experimental evidence into account. Lab-scale experiments focus on evaluating the physical and chemical impacts that dominate biofilm development and the pollutant degradation efficiency through imaging techniques and other analytical methods.

In addition to the lab-scale studies, field site monitoring of biofilm growth will provide complementary and unique information about the remediation project. However, the direct sampling method will either have little spatial resolution or be quite costly [15]. Nuclear magnetic resonance (NMR) can be applied to detect water molecules in a sample non-destructively and in situ. Furthermore, as the structural support of the biofilms, EPS consists of more than 95% water [8,16]. Hence, NMR is quite suitable for directly monitoring biofilm growth. The high-field NMR imaging and velocity-mapping have already been used to visualize biofilms and the related hydrodynamics and dispersion [17,18,19,20]. However, the high field strength gives rise to high magnetic susceptibility gradients inside the sample, restricting its application to porous geological media [15]. Consequently, low-field NMR, which can reduce the effects of magnetic susceptibility, is applied to study the bacterial fouling process in porous media. T_2_ relaxation measurements proved to be sensitive to the biofouling process not only in silica sand but also in natural unconsolidated sand with high magnetic material content [15]. Soon afterward, NMR well logging proved to be sensitive to biofilm formation procedures in a sand pack modeled well-bore environment [21] and an engineered filed testing site [7].

In a study by Alexis et al. [15], the reduction in the T_2_ relaxation was measured from both biofilm formation porous media and alginate gel solutions. As the alginate is from certain types of bacterial biofilms, the formation process of alginate gels was used by the researchers to represent the existence of biofilms. However, the measured relaxation could reduce with fluid characteristics such as viscosity and dissolved paramagnetic ions [22,23], and the biofilms not only consist of polysaccharides but also many proteins which also act as the structural support of the biofilm matrix [8]. It has already been discovered that the biofilm matrix is inhomogeneous; the physical and chemical structure of the matrix varies with fluid hydrodynamics and nutrient content [24,25,26]. This means that the water molecule distribution bounded in the network of biofilm matrix will change under different circumstances. Can the T_2_ distribution reflect the structure information of the biofilm matrix? How can we determine the peak that represents biofilms in the obtained low-field T_2_ distribution? The existing studies provide little detail about such information.

Hence, the objectives of this study are to (a) determine the mechanisms whereby the biofilm development process contributes to the low-field proton-proton relaxation signals and (b) assess the ability of T_2_ measurements to monitor the biofouling process.

## 2. Materials and Methods

### 2.1. Porous Media

In this study, to determine biofilms’ contributions to the T_2_ relaxation time of the biofilm formation in porous media, influences such as paramagnetic and magnetic impurities were not taken into account. The quartz sands, which were chosen for porous media, are analytically pure (chloride ≤ 0.015%, Fe ≤ 0.005%). The grain size ranged from 260 μm to 300 μm in diameter. Before the cultivation experiment, the SiO_2_ sands were immersed in diluted hydrochloric acid (10% in volume) for more than one hour to remove paramagnetic materials, then washed more than six times with deionized water until the PH was close to 7.0. Then, the quartz sands were dried in an oven overnight. The pore volume of pores between 17 Å and 3000 Å in diameter, which was evaluated by a Micromeritics ASAP 2020 Accelerated Surface Area and Porosimetry System, is 0.000535 cm³/g.

### 2.2. NMR Measurement Parameters

In an NMR measurement system, after applying an external oscillating perturbation to the ^1^H nuclear spins in the sample with water, the spins will then relax to the equilibrium state [27,28]. This detectable relaxation process can be characterized by the longitudinal relaxation time (T_1_) and the transverse relaxation time (T_2_). The initial amplitude is proportional to the total number of ^1^H spins presented in the water [28]. The distribution of the transverse relaxation time (T_2_) is less time-consuming and can be used to provide information about the fluid and the environment in which the fluid exists.

The NMR transverse relaxation times were collected by a 20-MHz NMR Instrument (Mini MR60) using a Carr–Purcell–Meiboom–Gill (CPMG) pulse sequence at 32 °C (the operation temperature of the instrument). The echo number is 18,000, and the echo time (t_E_) is 1000 μs. All the samples were measured under the same parameters to ensure consistency of experimental data sets. All the obtained relaxation times were normalized by the initial amplitude, and the inversed distributions were normalized by the total signal amplitude.

### 2.3. Bacterial Cultivation and Biofilm Cultivation

With the intention of in situ NMR measurement, the biofilm formation system was treated as a complete object which could not be destroyed. It is difficult to determine the influence of each process on the transverse relaxation, as well as the liquid media and the porous media. Therefore, we investigated T_2_ signals of the bacterial cell growth process, the biofilm growth process in the liquid media and in the liquid-filled porous media, respectively.

In this study, *Pseudomonas aeruginosa* was used as it is a contamination-degrading bacterium and can easily form biofilms [29,30]. Before cultivation, the LB (5 g/L yeast extract powder, 10 g/L NaCl, 10 g/L tryptone, pH = 7.4) culture media were sterilized by autoclaving at 120 °C for 20 min, the quartz sand was sterilized in a draught drying cabinet at 160 °C for 2 h, and the glass sample containers and other laboratory appliances were sterilized with a 75% (*v*/*v*) alcohol solution. After sterilization, all the materials (except for LB liquid media) were dried and exposed to ultraviolet radiation for more than one hour in the ultra-clean workbench for later use.

**Figure 1:**
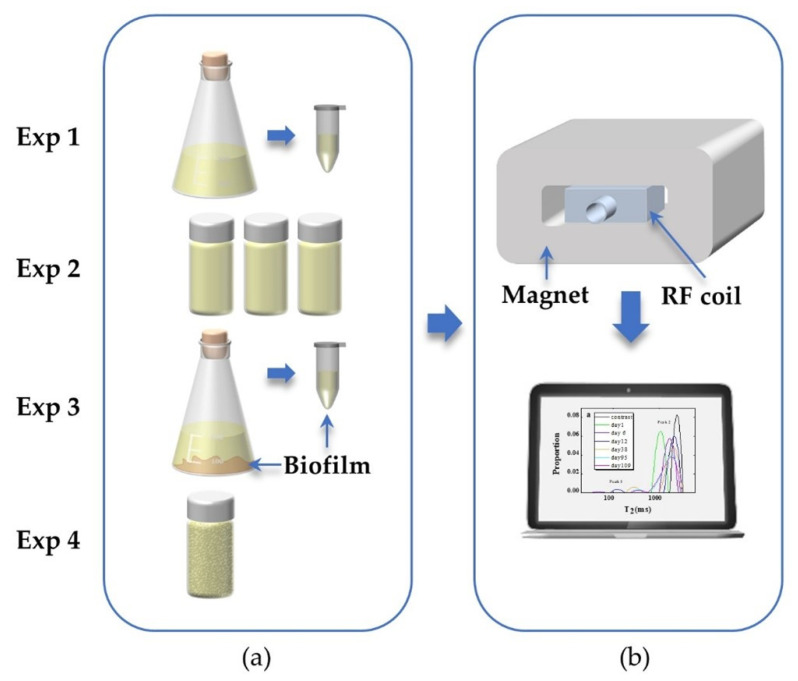
Schematic of (**a**) bacterial samples for the four experiments and (**b**) NMR measurement and data analysis unit.

Figure 1 shows the schematic map of the sample and the NMR measurement system. In experiment 1, two parallel samples (sample 1 and sample 2) with 200 mL sterile LB in triangular flasks were inoculated with 6 mL inoculum and incubated in a shaker (220 rmp, 37 °C). Every two hours, the samples were taken from the two flasks for the optical density (OD) measurement through an ultraviolet spectrophotometer (wavelength: 600 nm) and T_2_ measurement. In experiment 2, the bacteria cells were incubated overnight in a shaker. Then, the bacterial suspensions were centrifuged and re-suspended in sterile nutrient media; the cell concentrations were 50%, 100%, and 200% in contrast to the concentration before centrifuging. Then, 20 mL bacterial suspension in each concentration (b1, b2, and b3) was immediately removed to the transparent glass sample bottle. A contrast sample with 20 mL sterilized LB was also prepared. The samples were statically cultivated in a constant temperature incubator (32 °C). In experiment 3, a flask with 200 mL LB was inoculated with 6 mL inoculum and incubated overnight in a shaker, then re-suspended with 200 mL LB in a flask. The flask sample was statically cultivated in a constant temperature incubator (32 °C) for one week. Two samples were collected from the cultivated biofilms. Then, these two samples were taken for T_2_ measurements. After the NMR experiment, both samples were put into a −80 °C freezer before vacuum freeze-drying. In experiment 4, silica sand-filled glass bottles were used to model homogeneous porous media. In this way, the NMR characteristic of the porous media’s pore structures was consistent. Two porous media samples were prepared; one was saturated with sterilized LB as a contrast, and the other one was saturated with re-suspended bacterial suspension to ensure inoculation throughout the sample holder. Both samples were cultured in a constant temperature incubator (32 °C) during this biofilm formation experiment.

### 2.4. Identification of Biofilm Formation

In experiment 3, to determine the pore structure of the formation biofilm matrix, the biofilms were produced by vacuum freeze-drying. In experiment 4, the sands with biofilms were submerged in a 2.5% glutaraldehyde solution at 4 °C for 8–10 h, washed three times with a saline solution, and then dehydrated with ethanol (10%, 30%, 50%, 70%, 90%, and 100%, 10 min). All the samples were treated by platinum sputtering. Then, the samples were photographed under a Scanning Electron Microscope (SEM) (JSM-IT500).

## 3. Results

### 3.1. Growth Curve for Bacterial Cells

**Figure 2:**
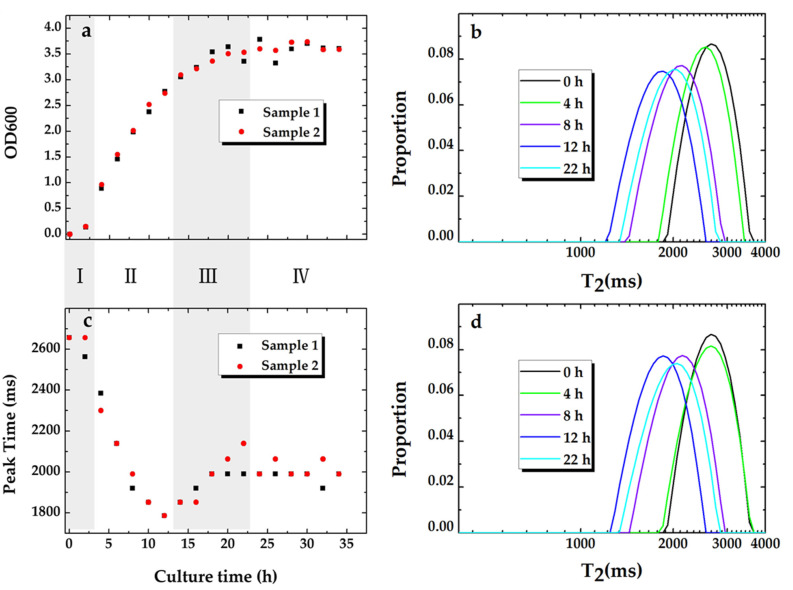
Growth curve of bacterial cells suspended in liquid media: (**a**) OD value vs. culture time; (**c**) the corresponding peak time of T_2_ relaxation distribution. The first points in (**a**,**c**) are measurements for the sterile liquid media before inoculation at room temperature. T_2_ relaxation distribution of the cell suspensions sampled at 0 h, 4 h, 8 h, 12 h, and 22 h for sample 1 (**b**) and sample 2 (**d**).

The OD value of the bacterial suspension is directly correlated to the cell concentration [31,32,33] and can be used as an indicator for the bacterial cells’ growth pattern. The culture time versus the OD values and the peak times of the T_2_ relaxation time distribution for the samples are presented in Figure 2a,c, respectively. The growth curves of the *P. aeruginosa* cells in Figure 2a show the whole bacterial growth periods (i.e., (I) the lag period, (II) the logarithmic period, (III) the stationary period, and (IV) the decline period). The curve trends for the two parallel samples are consistent. The corresponding peak time curves in Figure 2c also show a similar trend. The peak times sharply decrease at period II, increase at period III, and fluctuate within a narrow range at period IV. T_2_ relaxation distribution for the samples (sample 1 and sample 2) at 0 h, 4 h, 8 h, 12 h, and 22 h are plotted in Figure 2b,d. The distributions of bacterial cell suspensions behave as unimodal.

Fluctuations, which are observed in stage III and stage IV, correspond to the observation of granule groups in the culture media. These agglomerates are formed by a large number of bacterial cells, which result in the inhomogeneity of the culture media and increase the difficulty of sampling. The fluctuations of the experimental data are considered to be within a reasonable range.

### 3.2. Biofilm Formation in Fluid

**Figure 3:**
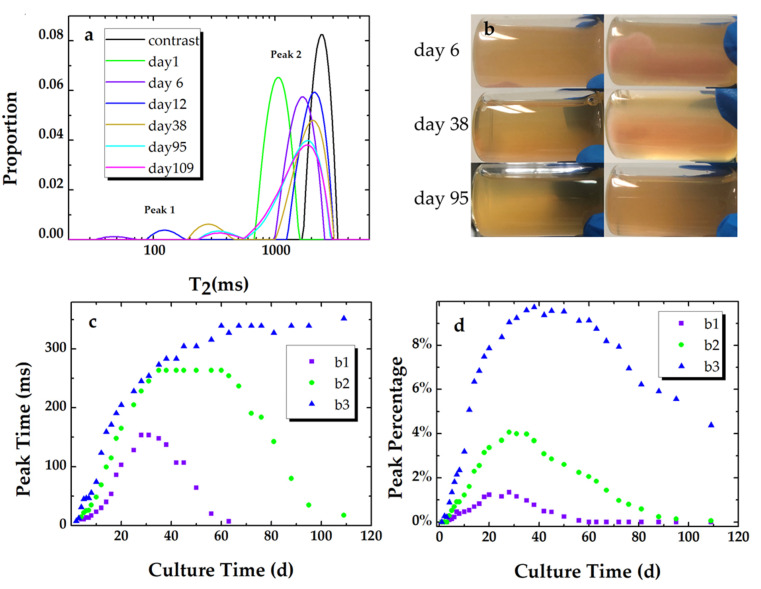
(**a**) T_2_ relaxation distributions of sample 3 (b3) at day 1, day 6, day 12, day 38, day 95, day 109, and a contrast sample. (**b**) Photos of sample 3, which were taken at day 6, day 38, and day 95 from a vertical insight (the row at left) and the bottom of the sample (the row at right). The peak times (**c**) and the corresponding peak percentage (**d**) of the T_2_ relaxation distributions for peak 1 of each sample (b1, b2, and b3) vs. culture time.

T_2_ relaxation distributions of sample 3 in experiment 2 at day 1, day 6, day 12, day 38, day 95, and day 109 are presented in Figure 3a. As seen in Figure 3a, the shape of the T_2_ spectrum evolves from unimodal (day 1) to bimodal. The bimodal T_2_ relaxation distribution is composed of a fast relaxation component (peak 1) and a slow relaxation component (peak 2). Studies have shown that the fast relaxation components that range from tens of milliseconds to hundreds of milliseconds are assigned to the biofilm phase [17,34,35]. Peak 1 and peak 2 are assigned as the biofilm phase and the bulk phase in this study, respectively. The difference in relaxation times for the biofilm phase can be attributed to the difference in biofilm density, which varied with the nutrient structure, nutrient concentration, and fluid flow condition [25,26,36].

Biofilm formation can be clearly identified at the bottom of the cylindrical glass sample, which is shown in Figure 3b. The volume of the formed biofilm increases while the average density appears to decrease. In addition, the boundary between the biofilm and the bulk becomes indistinct with the biofilm evolution process. The bulk (bacterial suspension) evolves from opaque to transparent and then returns to semitransparent, which indicates that the concentration of the suspended bacterial cells decreases and re-increases. In fact, the biofilm in each sample did not entirely disappear at the end of our experiment.

The peak times of the fast component (Figure 3c, peak 1) increase with culture time and reach maximum values. After several stationary days, the peak times persistently decreased except for sample 3. The percentage of peak 1 (Figure 3d) for each sample experienced a similar trend. For sample 3, though the decrease in the peak time of the biofilm phase was not measured, the decrease in the corresponding peak percentage had already started on day 40. In addition, the broad distribution of peak 2 for sample 3 shows a merging of an additional peak (T_2_ ~ 700 ms) and the bulk (T_2_ ~ 1900 ms). This phenomenon is attributed to nutrient exhaustion and the dissolution of the EPS matrix, which will cause quite a low density of the matrix. 1D T_2_ measurement is thus supposed to be unable to detect the existence of the low-density biofilm. In addition, the differences in the maximum values imply that a higher concentration of inoculum cells gives rise to more biofilm formation under static cultivation.

**Figure 4:**
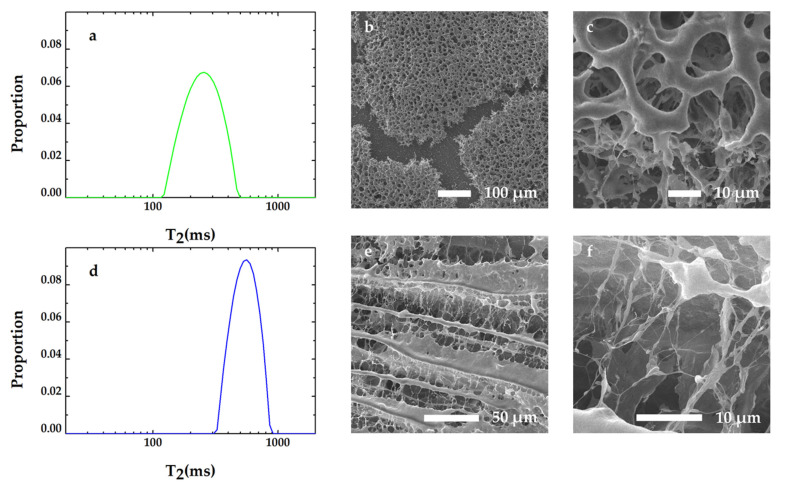
(**a**) T_2_ distribution of biofilm, which was obtained from the bottom of the cultured biofilm. (**b**,**c**) SEM images of biofilm corresponding to T_2_ in (**a**). (**d**) T_2_ distribution of biofilm, which was obtained from the upper layer of the cultured biofilm. (**e**,**f**) SEM images of biofilm corresponding to T_2_ in (**d**).

T_2_ distributions of the biofilms in experiment 3 are shown in Figure 4a,d. These two samples, which were obtained from different parts of the cultured biofilm, exhibited diverse T_2_ distributions. One (Figure 4a) displays a shorter relaxation time and a broader T_2_ distribution; the other one (Figure 4d) displays a longer relaxation time and a narrower T_2_ distribution. The SEM images corresponding to Figure 4a,d are shown in Figure 4b,c,e,f, respectively. Biofilms after vacuum freeze-drying in Figure 4b have connecting pores and smaller pore sizes, while biofilms after vacuum freeze-drying in Figure 4d have lamellose structure and larger pore structures. The cracks in Figure 4b,c are were supposed to generate during the sample-preparation process before its SEM image was taken. It is interesting that the bacterial cells connect to each other by pili-like nanowires at the broken site and embed in the EPS matrix in Figure 4b,c, while bacterial cells and the pili-like nanowires also exist in the space between the layers of the EPS matrix in Figure 4d,e. The results of experiment 3 also show that a larger space within the biofilm matrix (lower biofilm density) contributes to a slower T_2_.

### 3.3. Biofilm Formation in Porous Media

**Figure 5:**
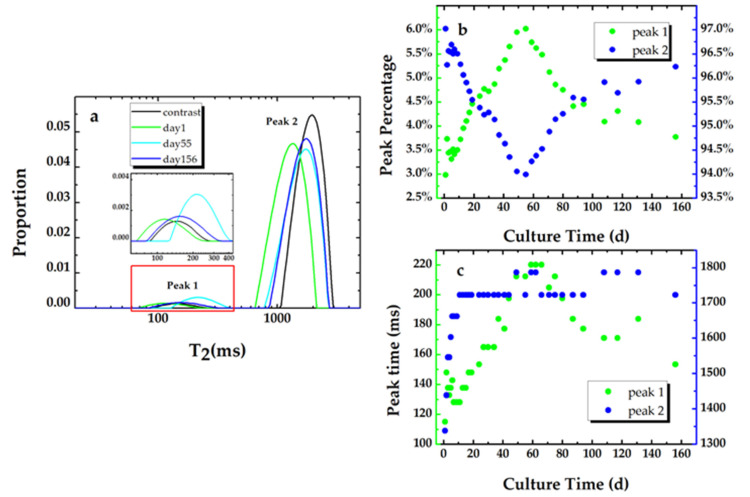
(**a**) The T_2_ relaxation distributions for the inoculated sample at day 1, day 55, and day 156 and the contrast sample. The peak percentage (**b**) and the peak time (**c**) of the T_2_ relaxation distribution for the biofouling sample.

**Figure 6:**
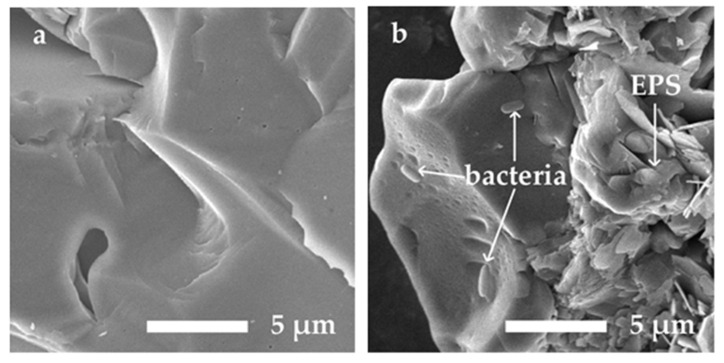
Scanning Electron Microscope (SEM) images of (**a**) the clean sands and (**b**) sand coated by EPS and bacterial cells.

In water-filled porous media, the T_2_ distribution gives the pore size distribution within the media [37,38,39]. In this study, the T_2_ distribution of the sand modeled porous media (the contrast sample in Figure 5a) that are saturated with sterile LB nutrients consist of two types of pore size distribution: the fast component and the slow component, which indicates the distributions of the mesopore and the macropore within the sand filled sample. The existence of the mesopore in the silica sands is confirmed by a BET (specific surface area) measurement. The macropore can be clearly identified in the SEM image (Figure 6a) of the clean sand sample. The space between the sand grains also constructs the macropore within the sand modeled porous media.

Figure 5a shows the T_2_ relaxation distributions for the inoculated sample at day 1, day 9, day 55, and day 156 and the contrast sample. The peak percentage and the peak time for both peak 1 (the fast component) and peak 2 (the slow component) vs. the culture time are plotted in Figure 5b,c, respectively. The pore size distribution of the sand pack is unchangeable, so it is suggested that the variation in the peak percentage is caused by the development of the biofilm phase. The proportion of the two peaks for the contrast sand pack saturated with sterile nutrients are 2.4% and 97.6%, respectively. As shown in Figure 5b, after the second sand pack is saturated with the bacterial suspension, the percentage of the fast component continues to increase and reaches a maximum value of 6% at day 56. The increase in the peak time of the fast component implies the increase in the average pore size of the mesopore within the sample. The increase in the proportion of the fast component implies the formation of the biofilm. Due to nutritional limitations, the maximum value was only kept for several days and then started to decrease and reached 3.7% at the end of the experiment, which indicates the biofilm dissolution results from the nutrient depletion.

The trend of the percentage changes of the culture time for peak 1 is consistent with the result of experiment 2. The peak time of the slow component increases and then keeps stable, which reveals that the bacterial cells that were suspended in the pore space attached to the pore surface. We can also observe that the peak time for the slow component did not return to the value for the sterilized one (the contrast sample) throughout the experiment. This indicates that there are still some bacterial cells suspended in the fluid within the pore spaces.

At the end of experiment 4, the formation of biofilm was confirmed by taking SEM images for the sands from the contrast sample (Figure 6a) and the biofilm formation sample (Figure 6b), respectively. The surface of the clean sand is smoother than that of the biofilm-coated sand. Bacterial cells and the EPS fragments can be clearly seen in Figure 6b.

## 4. Discussion

To determine the mechanisms whereby the biofilm development process contributes to the low-field T_2_ relaxation, four different *P. aeruginosa* cultivation experiments were conducted in the liquid media and porous media.

The results in experiment 1 indicate that the transverse relaxation is related to bacterial growth, especially in the logarithmic period when the bacterial cells are rapidly proliferating. The relaxation rate is supposed to be accelerated by cell proliferation. Furthermore, the water in the microbial cell is reported to be almost completely restricted by the cell wall [40]; it cannot be detected by one-dimensional, low-field NMR. Consequently, the bacterial cells suspended in the water are considered to be tiny particles that can accelerate proton–proton relaxation but do not contribute to the detectable amount of ^1^H protons in the fluid. This means that the one-dimensional transverse relaxation can reflect the concentration of bacterial cells but cannot detect bacterial cells themselves. We noticed that the peak time appeared to be increasing slightly from period III, when the total number of bacterial cells was still growing. This phenomenon is supposed to be induced by bacterial death and nutrient reduction during the later cultivating process. The transverse relaxation time shows a dependence on the density of the bacterial cells suspended in the nutrient liquid. The growth curve represented by the peak time of the T_2_ relaxation time distribution of the suspension can reflect the overall growth trend of bacterial cells in the suspension.

It is well known that the biofilm development processes include three stages [41,42,43]: stage 1, attachment and proliferation; stage 2, biofilm growth; stage 3, detachment. The result with regard to the change in the peak time and peak percentage of the T_2_ distribution for each sample in experiment 2 is consistent with the biofilm development process. In stage 1 and stage 2 of experiment 2, some bacterial cells attach to the surface of the glass sample holder, multiply, and secrete EPS. Other bacterial cells suspended in the nutrient solution also attach to the EPS matrix. In addition, in the two stages, as the metabolism procedure, bacteria will consume the nutrients and capture water molecules from the bulk, which will reduce the concentration of the nutrients and the bacterial cells in the bulk. Therefore, on the one hand, with the EPS secretion, a part of water molecules are bound to the biofilm matrix. The percentage and relaxation time for peak 1 increase with the growth of biofilm. On the other hand, the relaxation time and the peak time of the bulk water increase (not shown in detail) with the attachment of bacterial cells to the EPS matrix in the suspension, which can be verified from the phenomenon that the turbidity of the bacterial cells’ suspension decreased with time. It can be inferred that the bulk relaxation time increases with the concentration decrease of the bacterial cells suspended in the bulk, which is also consistent with the result of experiment 1. In stage 3, biofilm growth stopped due to nutrient limitations and the consumption of EPS by bacterial cells in that the nutrients were depleted and the bacterial cells partially detached into the water. Correspondently, the percentage and relaxation time of peak 1 were reduced. An independent measurement of samples with biofilm in experiment 3 also proves that the fast component represents the biofilm phase and gives information about the biofilm matrix structure.

According to the result in experiment 4, the change of the relaxation time for the fast component can be attributed to the difference in the biofilm density formed in the sample’s pore space, since there are neither paramagnetic metallic ions in the nutrient nor metallic minerals in the silica sands. Therefore, the formed biofilm can act as an additional porous media in the biofouling porous system in analyzing the measured data set, while the relaxation time of the slow component can be ascribed to the density of the bacterial cells in the larger pores and the pores between the sand grains. The results indicate that the non-invasive T_2_ relaxation measurement can provide information for the biofilm development process in porous media.

In conclusion, a low-field NMR T_2_ measurement can provide information such as the microbial cells’ concentration, the formation of the biofilm phase, and the fate of the biofilm phase. It is possible to detect the biofilm formation, monitor the development process of biofilm in bulk water, and obtain information on the development process of biofilm in porous media. However, biofilm with a low density, which may be induced by nutrient exhaustion, may not be detected by 1D T_2_ measurement. The results of this experiment provide opportunities for low-field NMR to detect the existence of biofilm in pure fluid environments such as water pipes and water tanks. The biofilm cultivation experiments in this paper were only conducted under static conditions in which nutrient contents were limited. More attention should be paid to the flow conditions, which can provide a more comprehensive interpretation of the monitoring data.

## Data Availability

Not applicable.
